# Supplementary material for: Assessment of dream-related aspects and beliefs in a large cohort of French students using a validated French version of the Mannheim Dream questionnaire
Source: PLoS One. 2021 Mar 4;16(3):e0247506. doi: 10.1371/journal.pone.0247506 (PMC7932137; doi:10.1371/journal.pone.0247506)
Supplement: S1 Table — (DOCX) [file pone.0247506.s002.docx]

**S1 Table. Description of emotional dream variables distribution of the total sample stratified by gender**

|  | | **Gender** | | | |  |  |
| --- | --- | --- | --- | --- | --- | --- | --- |
|  |  | **Women (n = 923)** | | **Men (n = 214)** | | **Total (N = 1137)** | |
| *Categorial Variables* | | **n** | **%** | **n** | **%** | **n** | **%** |
| **Emotional intensity** | |  |  |  |  |  |  |
|  | Not at all intense | 9 | 0,98 | 7 | 3,27 | 16 | 1,41 |
|  | Not that intense | 77 | 8,34 | 27 | 12,62 | 104 | 9,15 |
|  | Somewhat intense | 294 | 31,85 | 67 | 31,31 | 361 | 31,75 |
|  | Quite intense | 428 | 46,37 | 90 | 42,06 | 518 | 45,56 |
|  | Very intense | 115 | 12,46 | 23 | 10,75 | 138 | 12,14 |
| **Emotional tone** | |  |  |  |  |  |  |
|  | Very negative | 67 | 7,26 | 11 | 5,14 | 78 | 6,86 |
|  | Somewhat negative | 349 | 37,81 | 57 | 26,64 | 406 | 35,71 |
|  | Neutral | 248 | 26,87 | 79 | 36,92 | 327 | 28,76 |
|  | Somewhat positive | 220 | 23,84 | 47 | 21,96 | 267 | 23,48 |
|  | Very positive | 39 | 4,23 | 20 | 9,35 | 59 | 5,19 |
