# Supplementary material for: Assessment of dream-related aspects and beliefs in a large cohort of French students using a validated French version of the Mannheim Dream questionnaire
Source: PLoS One. 2021 Mar 4;16(3):e0247506. doi: 10.1371/journal.pone.0247506 (PMC7932137; doi:10.1371/journal.pone.0247506)
Supplement: S2 Table — (DOCX) [file pone.0247506.s003.docx]

**S2 Table. Distributions of current nightmares, childhood nightmares and lucid dreams in the total sample stratified by gender.**

|  | | **Gender** | | | |  |  |
| --- | --- | --- | --- | --- | --- | --- | --- |
|  |  | **Women (n = 923)** | | **Men (n = 214)** | | **Total (N = 1137)** | |
| *Categorial Variables* | | **n** | **%** | **n** | **%** | **n** | **%** |
| **Current nightmare frequency** | |  |  |  |  |  |  |
|  | Never | 27 | 2,93 | 21 | 9,81 | 48 | 4,22 |
|  | Less than once a year | 30 | 3,25 | 24 | 11,21 | 54 | 4,75 |
|  | About once a year | 54 | 5,85 | 22 | 10,28 | 76 | 6,68 |
|  | About two to four times a year | 181 | 19,61 | 52 | 24,30 | 233 | 20,49 |
|  | About once a month | 177 | 19,18 | 39 | 18,22 | 216 | 19,00 |
|  | Two to three times a month | 216 | 23,40 | 30 | 14,02 | 246 | 21,64 |
|  | About once a week | 145 | 15,71 | 15 | 7,01 | 160 | 14,07 |
|  | Several times a week | 93 | 10,08 | 11 | 5,14 | 104 | 9,15 |
| **Childhood nightmare frequency** | |  |  |  |  |  |  |
|  | Never | 21 | 2,28 | 10 | 4,67 | 31 | 2,73 |
|  | Less than once a year | 25 | 2,71 | 11 | 5,14 | 36 | 3,17 |
|  | About once a year | 41 | 4,44 | 10 | 4,67 | 51 | 4,49 |
|  | About two to four times a year | 162 | 17,55 | 48 | 22,43 | 210 | 18,47 |
|  | About once a month | 209 | 22,64 | 41 | 19,16 | 250 | 21,99 |
|  | Two to three times a month | 215 | 23,29 | 45 | 21,03 | 260 | 22,87 |
|  | About once a week | 161 | 17,44 | 25 | 11,68 | 186 | 16,36 |
|  | Several times a week | 89 | 9,64 | 24 | 11,21 | 113 | 9,94 |
| **Lucid dream frequency** | |  |  |  |  |  |  |
|  | Never | 221 | 23,94 | 60 | 28,04 | 281 | 24,71 |
|  | Less than once a year | 73 | 7,91 | 19 | 8,88 | 92 | 8,09 |
|  | About once a year | 51 | 5,53 | 15 | 7,01 | 66 | 5,80 |
|  | About two to four times a year | 117 | 12,68 | 39 | 18,22 | 156 | 13,72 |
|  | About once a month | 116 | 12,57 | 25 | 11,68 | 141 | 12,40 |
|  | Two to three times a month | 116 | 12,57 | 18 | 8,41 | 134 | 11,79 |
|  | About once a week | 132 | 14,30 | 9 | 4,21 | 141 | 12,40 |
|  | Several times a week | 97 | 10,51 | 29 | 13,55 | 126 | 11,08 |
