# Supplementary material for: Assessment of dream-related aspects and beliefs in a large cohort of French students using a validated French version of the Mannheim Dream questionnaire
Source: PLoS One. 2021 Mar 4;16(3):e0247506. doi: 10.1371/journal.pone.0247506 (PMC7932137; doi:10.1371/journal.pone.0247506)
Supplement: S3 Table — (DOCX) [file pone.0247506.s004.docx]

**S3 Table. Distribution of responses for items on question 12 “Attitude towards dreams”**

|  | Not at all | | Not that much | | Partly | | Somewhat | | Totally | |
| --- | --- | --- | --- | --- | --- | --- | --- | --- | --- | --- |
| ***Categorial Variables*** | **n** | **%** | **n** | **%** | **n** | **%** | **n** | **%** | **n** | **%** |
| **A- How much meaning do you attribute to your dreams?** | 107 | 9,41 | 255 | 22,43 | 296 | 26,03 | 345 | 30,34 | 134 | 11,79 |
| **B- How strong is your interest in dreams?** | 56 | 4,93 | 180 | 15,83 | 226 | 19,88 | 378 | 33,25 | 297 | 26,12 |
| **C- I think that dreams are meaningful** | 31 | 2,73 | 86 | 7,56 | 220 | 19,35 | 427 | 37,55 | 373 | 32,81 |
| **D- I want to know more about dreams** | 29 | 2,55 | 42 | 3,69 | 93 | 8,18 | 280 | 24,63 | 693 | 60,95 |
| **E- If somebody can recall and interpret his/her dreams. his/her life will be enriched** | 59 | 5,19 | 185 | 16,27 | 291 | 25,59 | 358 | 31,49 | 244 | 21,46 |
| **F- I think that dreaming is in general a very interesting phenomenon** | 5 | 0,44 | 18 | 1,58 | 50 | 4,40 | 272 | 23,92 | 792 | 69,66 |
| **G- A person who thinks about her/his dreams is certainly able to learn more about her/himself** | 14 | 1,23 | 76 | 6,68 | 195 | 17,15 | 427 | 37,55 | 425 | 37,38 |
| **H- Do you have the impression that dreams provide dreams provide impulses or pointers for your waking life?** | 94 | 8,27 | 238 | 20,93 | 308 | 27,09 | 330 | 29,02 | 167 | 14,69 |
