# Supplementary material for: Assessment of dream-related aspects and beliefs in a large cohort of French students using a validated French version of the Mannheim Dream questionnaire
Source: PLoS One. 2021 Mar 4;16(3):e0247506. doi: 10.1371/journal.pone.0247506 (PMC7932137; doi:10.1371/journal.pone.0247506)
Supplement: S4 Table — (DOCX) [file pone.0247506.s005.docx]

**S4 Table. Frequency distribution of different effect of dreams on waking life**

| Categorial Variables | **13. Telling dreams** | | **14. Recording dreams** | | **15. Dreams affecting daytime mood** | | **16. Dreams providing creative ideas** | | **17. Dreams solving problems** | | **18. Déjà-vu** | |
| --- | --- | --- | --- | --- | --- | --- | --- | --- | --- | --- | --- | --- |
|  | **n** | **%** | **n** | **%** | **n** | **%** | **n** | **%** | **n** | **%** | **n** | **%** |
| Never | 59 | 5,19 | 886 | 77,92 | 301 | 26,47 | 581 | 51,10 | 473 | 41,60 | 44 | 3,87 |
| Less than once a year | 47 | 4,13 | 63 | 5,54 | 102 | 8,97 | 120 | 10,55 | 129 | 11,35 | 46 | 4,05 |
| About once a year | 45 | 3,96 | 31 | 2,73 | 69 | 6,07 | 92 | 8,09 | 100 | 8,80 | 61 | 5,36 |
| About two to four times a year | 145 | 12,75 | 54 | 4,75 | 180 | 15,83 | 131 | 11,52 | 185 | 16,27 | 222 | 19,53 |
| About once a month | 211 | 18,56 | 32 | 2,81 | 157 | 13,81 | 95 | 8,36 | 100 | 8,80 | 222 | 19,53 |
| Two to three times a month | 247 | 21,72 | 34 | 2,99 | 135 | 11,87 | 64 | 5,63 | 99 | 8,71 | 278 | 24,45 |
| About once a week | 235 | 20,67 | 19 | 1,67 | 115 | 10,11 | 31 | 2,73 | 35 | 3,08 | 151 | 13,28 |
| Several times a week | 148 | 13,02 | 18 | 1,58 | 78 | 6,86 | 23 | 2,02 | 16 | 1,41 | 113 | 9,94 |
