# Supplementary material for: Assessment of dream-related aspects and beliefs in a large cohort of French students using a validated French version of the Mannheim Dream questionnaire
Source: PLoS One. 2021 Mar 4;16(3):e0247506. doi: 10.1371/journal.pone.0247506 (PMC7932137; doi:10.1371/journal.pone.0247506)
Supplement: Attachment — Submitted filename: Response to reviewers.docx [file pone.0247506.s006.docx]

January 24^th^, 2021

Serena Scarpelli

Academic Editor

*PlosOne*

Dear Ms. Scarpelli,

We are pleased to submit this revised version of our manuscript entitled, “Assessment of dream-related aspects and beliefs in a large cohort of French students using a validated French version of the Mannheim Dream questionnaire” for consideration by *PlosOne.* We would like to thank the reviewers for their input and constructive comments that have helped us to substantially improve this manuscript. Please find below a summary of these comments as well as our responses to them and a description of subsequent changes to the text.

Data Availability statement: After verification with the authorities in our country, we are happy to be able to modify our data availability statement in order to make the dataset available (as is now noted in the Supporting Information section).

**Replies to reviewers:**

**Reviewer 1:**

Comment: “*Research questionnaire: given the centrality of the questionnaire on which all results are based, a more detailed description of the measure is needed. It might be helpful to label every point of response scales (here you may find an example:* [*https://doi.org/10.1007/s11818-019-0199-3*](https://doi.org/10.1007/s11818-019-0199-3)*). Moreover, some items are completely missing in the description (e.g., distress evaluation of nightmares or déjà vu experience).*”

Response: As requested by the reviewer and in order to improve the readability of the manuscript, we have added additional information about the MADRE questionnaire along with a reference to an online version of the validated French questionnaire (appendix of Ghorayeb et al., 2019). The description of this instrument is now thorough, with items labeled according to the reviewer’s request (Research Questionnaire paragraph, page 5/6, line 103 to 136).

Comment: “*Study population: the self-selection recruitment of studies based on online surveys may represent a limit in the interpretation of results, and this should be mentioned in the discussion.*”

Response: We fully agree with the reviewer’s comment that online surveys may limit the interpretation of results. We now discuss this limitation at the end of the Discussion section (page 18, line 383 to 387).

Comment: “*Population description: I would suggest moving this section in the previous section called “Study population”, for consistency and to improve readability.*”

Response: As suggested by the reviewer, we made this alteration in the revised manuscript by adding a section “Recruitment” and a section “Sample Description” to the “Study Population” paragraph (page 7, line 153 to 158).

Comment: “*Reporting of the data: the authors report a descriptive table (Table 1) of frequency distribution (stratified by gender) only in reference to “Dream recall frequency”. Otherwise, Table 2 presents the regression analysis only for “variables concerning dream’s effects on waking life”. I would suggest to upload the descriptive table of the frequency distribution for all items in a supplementary materials section, and adding a summary table for all regression analyses conducted.*”

Response: As recommended by the reviewer, we have added full descriptive tables in the Supporting Information section (see Tables S1 to S4 in the Supporting Information Section). As all regression analyses we conducted are detailed in the main text, we believe that adding *a summary table for all regression analyses conducted* would be redundant with findings already presented in the text. Should the reviewer feel differently after considering this point, we would be happy to make further alterations to the Supporting Information section.

Comment: “*Results: why the authors detailed results considering gender separately only with regard to “dream recall frequency” and “attitude toward dreams”? the authors should clarify the hypotheses on which the analyses are based*.”

Response: As many analyses were conducted in this study, we decided to analyze by gender only the most central variables for which sex differences have already been well-established (Schredl & Reinhard, 2008). Beyond these descriptions that we believe are useful for comparing our study with past investigations, we did not comprehensively pursue analyses by gender because of differences in sample size. To go further in this direction would require us to collect new data with more male students. However, following the reviewer’s comment, this issue is now raised as a limitation in the Discussion section (page 17, line 352 to 353).

Comment: “*There seems to be different font sizes used throughout the manuscript (e.g. line 90 and line 114)*” and “*Please ensure that you have this paper language edited and proofread (possibly by a native English speaker) before submitting the revised version.*”

Response: We thank the reviewer for pointing out these issues. The revised manuscript has been carefully proofread for formatting errors as well as for spelling mistakes and stylistic issues by a native English speaker.

**Reviewer 2:**

Comment: “*Introduction should talk more about what motivated the study. Why is it important to study dreams in a student population? What is the relevance of those finds in the light of the dream theories?* *Here I suggest looking at the Emotional Regulation Theory from Toren Nielsen.*”

Response: In light of the reviewer’s comment, we altered the Introduction to formulate more explicitly the general framework of the survey (page 3, line 57 to 59, and page 4, line 85 to 87). This population is of particular interest because of its homogeneity, in that students have a common lifestyle with similarities in their sleep patterns between weekdays and weekends. Nonetheless, we can identify differences in sub-groups of students, and we wanted to focus on this aspect to describe the dreams of students in a more detail. Before positioning our results relative to dream theory, we believe that an important first step is to better understand the relationships between sociodemographic characteristics and dream variables as well as their role in emotional regulation. We have therefore added information to the Introduction and Discussion sections to clarify that the main goal of this investigation is to describe a population, and not to test a particular theory. Such tests of theoretical models could be targeted in future studies, using the MADRE questionnaire along with other measures (e.g., relative to emotional regulation skills, or sleep quality and quantity) that would allow identification of factors that may explain dream recall frequency. Following the reviewer’s comments, we now note in the revised Discussion section of how prerequisite information on population characteristics and confounds would help future theory-based investigations, such as concerning Emotional Regulation Theory (page 15, line 306 to 308).

Comment: “*Page 6, line 142: while describing the population, it is crucial to describe the gender distribution in each scientific discipline, comparing the frequencies of male and female. It is not enough to only mention: "Women and men are equally distributed between the two fields of study". As the results are related to gender and scientific disciplines, this issue is essential to be precise.*”

Response: As request by the reviewer, we have added the number and percentages of men and women in each discipline to describe more precisely our sample (page 7, line 157 to 158).

Comment: “*The same population that reveals higher dream recall frequency (women and students from humanities) showed higher intensity in emotional tone. Are these results associated? This would be an interesting topic to explain the global phenomenon in this population. Women at the beginning of their academic careers might have higher academic stress, especially in STEMs. This could impact the emotional tone of their dreams, as well as the dream recall frequency.*”

Response: We thank the reviewer for raising this important point. With the results that we present in this article, it seems that women reported a higher dream recall frequency compared to men but that their dream recall was not different between humanities and science students. This lack of difference would tend to argue that higher dream recall frequency cannot easily be attributed specifically to women in the humanities. It would therefore be difficult to explore a relationship between these results and those observed on the intensity and tone of dreams, since we cannot know if participants reporting higher dream recall frequency are the ones reporting higher intensity or extreme dream tone. We nonetheless agree with the reviewer that the findings raise interesting questions in this regard that may be examined more directly in future investigations.

Comment: “*A better description of what was measured as "attitude toward dreams" before jumping to results can help a better understanding.*”

Response: As recommended by the reviewer, we added a short description of this variable in the Introduction (page 4, line 69 to 70).

Comment: “*Sleep disorder should be considered in the results (and even considered as a covariate). Did the authors collected any data related to sleep disorder? If not, this should be mentioned as a limitation.*”

Response: We fully agree with the reviewer that sleep disorders are an important issue to consider when possible. However, we did not collect data concerning these disorders or on sleep quality or quantity. As requested, we therefore raise this point as a limitation in the Discussion (page 15, line 317 to 318). Our objective here was to characterize dream characteristics and beliefs in a student sample; we did not consider other very numerous aspects that could potentially co-vary with these variables, and now state in the revised manuscript that such analyses should be considered in future research.

Comment: “*The discussion and the introduction should address more about dreams theories and their relationship with the academic path (pointing out what these results add to the field of dreams)*”

Response: We agree that this is an important issue, and we have also addressed this point in our response to the reviewer’s first comment. A large majority of studies in the area of dream research includes students as their population of study. Before addressing questions relevant to the function of dreams from a theoretical perspective, we wanted to first have a clearer image of what socio-demographic factors could influence dream variables among this particular population. For this reason, our results are not meant to directly test or compare different theoretical perspectives relative to dreams. We believe this would require a more specific survey design with additional instruments to address specific theoretical constructs. However, we nonetheless agree with the reviewer that theory-based research is an important goal and we hope that our manuscript contributes to this objective by providing lacking information on the population that is most studied in the field.

In summary, we would like to express our gratitude to both reviewers for their many constructive comments that helped us to substantially improve our manuscript. We hope that the present contribution is now acceptable for publication in *PlosOne*. Should the reviewers or Editor require any further modifications, we would be happy to respond rapidly to any additional suggestion.

Best wishes,

Imad Ghorayeb, M.D, Ph.D.

Department of Clinical Neurophysiology

CHU de Bordeaux Pellegrin,

33000 Bordeaux, France.

Email: [imad.ghorayeb@u-bordeaux.fr](mailto:imad.ghorayeb@u-bordeaux.fr)

**References:**

1. Ghorayeb I, Napias A, Denechere E, Mayo W. Validation of the French version of the Mannheim Dream Questionnaire in a French adult sample. 2019;12(2):12.
2. Schredl M, Reinhard I. Gender differences in dream recall: a meta-analysis. J Sleep Res. juin 2008;17(2):125‑31.
